# Association of maternal personality traits with medication use during pregnancy to appraise unmeasured confounding in long-term pharmacoepidemiological safety studies

**DOI:** 10.3389/fphar.2023.1160168

**Published:** 2023-05-15

**Authors:** Angela Lupattelli, Nhung T. H. Trinh, Hedvig Nordeng

**Affiliations:** ^1^ Pharmacoepidemiology and Drug Safety Research Group, Department of Pharmacy, Faculty of Mathematics and Natural Sciences, University of Oslo, Oslo, Norway; ^2^ Department of Child Health and Development, Norwegian Institute of Public Health, Oslo, Norway

**Keywords:** personality, prenatal medication use, unmeasured confounding, child development, E-value, perinatal pharmacoepidemiology

## Abstract

Maternal personality is a possible confounder on the association between prenatal medication exposure and long-term developmental outcomes in offspring, but it is often unmeasured. This study aimed to (i) estimate the association between five maternal personality traits and prenatal use of acetaminophen (including extended use), opioid analgesics, antidepressants, benzodiazepines/z-hypnotics, and antipsychotics; (ii) evaluate, using an applied example, whether unmeasured confounding by maternal neuroticism would make the association between prenatal antidepressant-child ADHD null, using the E-value framework. We used data from 8,879 pregnant women and recent mothers who participated in the Multinational Medication Use in Pregnancy Study, a web-based cross-sectional study performed within the period from 1-Oct-2011 to 29-Feb-2012 in Europe, North America and Australia. Medication use in pregnancy was self-reported by the women. Personality was assessed with the Big Five Inventory, capturing the dimensions of neuroticism, extraversion, openness, agreeableness, and conscientiousness. Adjusted logistic regression analyses were conducted for each trait-medication pair, using the survey weighting. There was a strong association between having high neuroticism and prenatal use of antidepressants (Odds Ratio (OR): 5.63, 95% Confidence Interval (CI): 3.96-8.01), benzodiazepines/z-hypnotics (OR: 6.66, 95% CI: 4.05-10.95), and analgesic opioids (OR: 2.24, 95% CI: 1.41-3.56), but not with antipsychotics. Among women with mental illness, this association attenuated for benzodiazepines/z-hypnotics, but decreased to the null for antidepressants. High neuroticism (OR: 1.31, 95% CI: 1.08-1.59) and high openness (OR: 0.77, 95% CI: 0.64-0.93) were associated with extended use of acetaminophen. The E-value for the Hazard Ratio 1.93 in the applied example was 3.27. If the example study was conducted using a population comparison group, high maternal neuroticism could have explained away the association antidepressant-ADHD. Because the example study included only women with a mental illness, this risk of bias was assessed as minimal. Various personality dispositions in the mother are associated, with a different degree, to prenatal use of medication. The strength of these association can aid researchers in evaluating the influence of uncontrolled confounding by maternal personality in long-term safety studies in pregnancy, using the E-value. This assessment should always be performed in addition to a rigorous study design using approaches to triangulate the evidence.

## 1 Introduction

Pregnant women are routinely excluded from pre-approval clinical trials, limiting our knowledge about risks and benefits of medication in this population and their offspring (Scaffidi et al., 2017). Pharmacoepidemiological pregnancy studies are valued as a methodological key to generate this needed evidence (Lupattelli et al., 2018a; Huybrechts et al., 2019). Their observational nature however entails inherent limitations and pitfalls, including unmeasured confounding, that need to be dealt with in order to generate sound, minimally biased risk measures (Lupattelli et al., 2018a; Huybrechts et al., 2019).

In the last 2 decades, there has been an important escalation of published data about the risk posed by prenatal medication exposure on offspring longer-term developmental outcomes (Hjorth et al., 2019). Multiple studies have investigated the neurotoxicity of acetaminophen and other analgesics, as well as of psychotropics (Hjorth et al., 2019; Sujan et al., 2019; Kwok et al., 2022; Wang et al., 2022). This research prioritization is important as 40%–60% of pregnant women use acetaminophen at least once during gestation (McKenna and McIntyre, 2006; Lupattelli et al., 2014), and the use of analgesic opioids has been increasing in some countries (Desai et al., 2014; Sujan et al., 2021). Antidepressants (estimate of use: 3%–8%) (Huybrechts et al., 2013; Molenaar et al., 2020), anxiolytics/sedatives and antipsychotics (estimate of use: 1%–5%) (Haas et al., 2018; Bais et al., 2020; Reutfors et al., 2020), although less common, constitute the treatment mainstay for maternal psychiatric disorders.

Establishing prenatal drug effects on child development using observational data is not simple. Unmeasured confounding by genetic liability, familial environment and other possible factors often hampers to draw any causal conclusion. Maternal personality is also an unmeasured confounder of interest in such studies; indeed, personality is an expression of partly genetically determined dispositions that influence the woman’s behaviour, and this correlate is linked to child development (Koutra et al., 2017). Recently, it has been shown that polygenic risk score for neuroticism remains associated with child attention-deficit/hyperactivity disorder (ADHD) even after adjusting for the child polygenic score, suggesting genetic nurture (Pingault et al., 2022). Maternal personality traits also associate with use of specific medications (e.g., paracetamol, sedatives/anxiolytics or antidepressants) in pregnancy (Ystrom et al., 2012), although data on this relationship are scarce. Maternal personality is possibly key confounder in longer-term safety studies on medications in pregnancy, yet it remains unmeasured in administrative or health registry data.

A more systematic use of bias analyses for unmeasured confounding, e.g., the E-value (Ding and VanderWeele, 2016; VanderWeele and Ding, 2017), has been advocated in epidemiological research. Briefly, the E-value technique requires that researchers loosely define the magnitude of the association of the possible unmeasured confounder with both exposure and outcome (e.g., BMI, alcohol intake), but these estimates are often unknown. This may challenge the overall interpretation of medication effects in light of the applied bias analyses for unmeasured confounding (Sjölander and Greenland, 2022).

The aim of this study was two-fold. We first quantified the association between five maternal personality traits and prenatal use of acetaminophen, analgesic opioids, antidepressants, antipsychotics, and anxiolytics and z-hypnotics. We selected these medication groups based on their prevalence of use in pregnancy and their necessity to treat important maternal conditions. Then, using one applied example, we assessed whether unmeasured confounding by the maternal personality trait neuroticism would make the associations between prenatal antidepressant exposure and child ADHD null, using the E-value framework (Ding and VanderWeele, 2016; VanderWeele and Ding, 2017). We choose ADHD outcomes and the personality trait neuroticism because these two factors are linked (Pingault et al., 2022), and because we recently have studies prenatal exposure to antidepressants and risk of ADHD (Lupattelli et al., 2021).

## 2 Materials and methods

### 2.1 Study design and data collection

**FIGURE 1:**
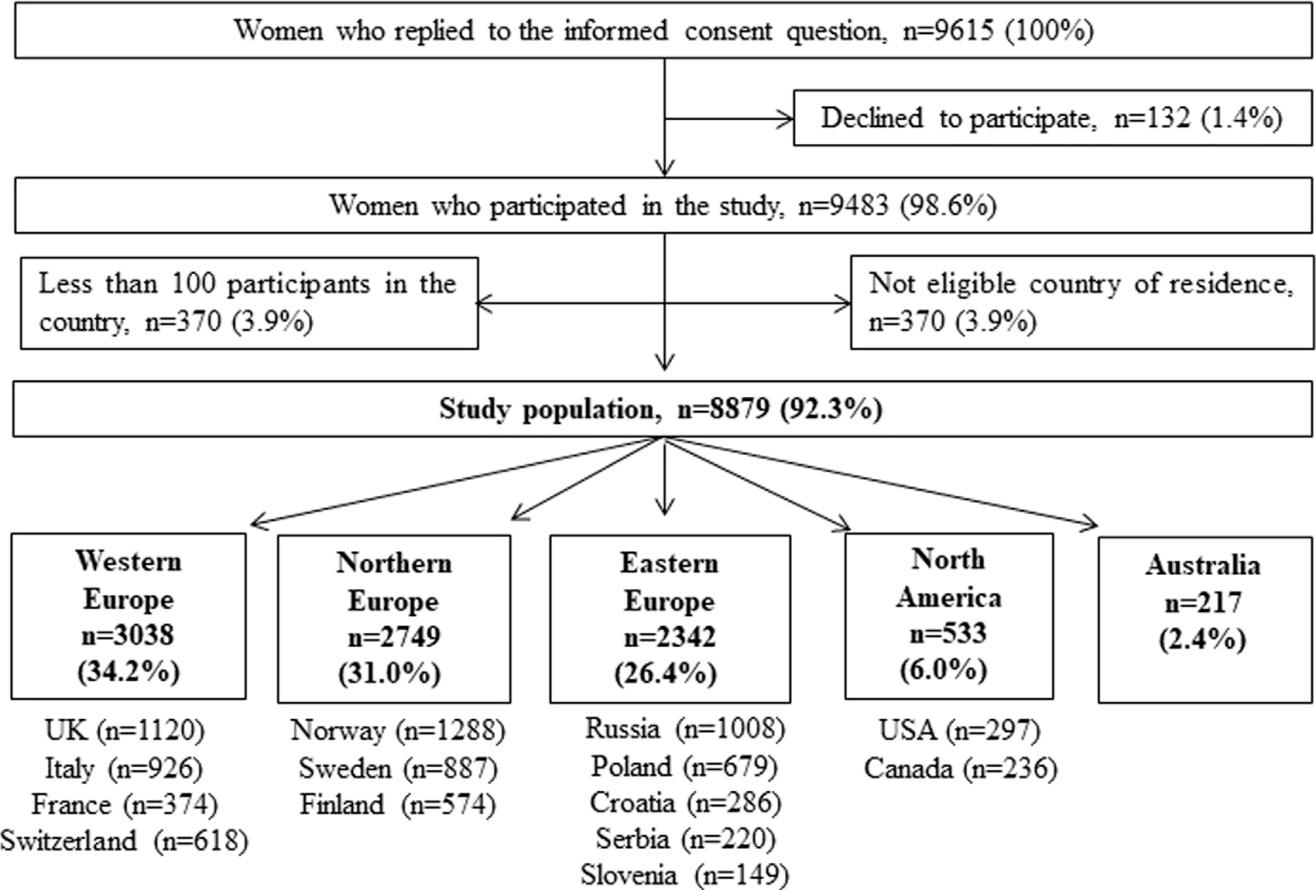
Data-flow to achieve the final study population.

This is a sub-study of the “*Multinational Medication Use in Pregnancy Study*” (Lupattelli et al., 2014), a multinational, cross-sectional, web-based investigation to examine patterns and correlates of medication use in pregnancy. Data were collected via a self-administered anonymous questionnaire (www.questback.com) in 18 countries. Pregnant women at any gestational week and mothers with a child less than 1 year of age could participate. Women located in Europe (i.e., Austria, Croatia, Finland, France, Iceland, Italy, the Netherlands, Norway, Poland, Russia, Serbia, Slovenia, Sweden, Switzerland, the United Kingdom (UK)), the United States, Canada and Australia were eligible to participate. The questionnaire also elicited responses from Central and South America, but due to the low numbers from each country in these regions, they were excluded from analysis. For further analysis, we excluded countries with less than 100 participants and aggregated the remaining ones into five regions: Western Europe (incl. Italy, France, Switzerland, the United Kingdom), Northern Europe (incl. Norway, Sweden, Finland), Eastern Europe (incl. Serbia, Slovenia, Russia, Poland), North America (incl. Canada and the United States), and Australia. The data selection process to achieve the final study sample of 8,879 women is illustrated in Figure 1.

The study was advertised on 2-3 pregnancy-related websites in each country, pregnancy forums and social media, and was open to the public for 2 months between October 2011 and February 2012 in each participating country. The recruitment national websites were selected for having the greatest number of daily users. The full questionnaire and further details about recruitment and the tools have been previously published (Lupattelli et al., 2014).

Informed consent was given by the participants by ticking the answer “yes” to the question “Are you willing to participate in the study?” The Regional Ethics Committee in Norway, region South-East, granted an ethical approval exemption for this study because of anonymity. Ethical approval or study notification to the relevant national Ethics Boards was achieved in the United Kingdom and Italy as required by the national legislation. All data were handled and stored anonymously.

### 2.2 Personality traits

Maternal personality traits were assessed with the Big Five Inventory (BFI) developed by John, Donahue and Kentle (John et al., 2008). The BFI measures the central elements of personality dimensions being extraversion, agreeableness, neuroticism, conscientiousness and openness. Participants were asked to rate the 44 statements on a scale from 1) disagree strongly to 5) agree strongly, which elicited different scores for each personality dimension. A comprehensive explanation of the statements and their scoring has been previously published (Lupattelli et al., 2018b). The extraversion and neuroticism dimensions included eight items each, making a score 8–40; agreeableness and conscientiousness included nine items, making a score 9–45; openness included ten items, with resulting score with range 10–50. Mean scores were calculated and then standardized (z-score) for each personality dimension. Higher z-scores indicate greater endorsement in the personality trait, and lower scores indicate the converse. To inform bias analysis for unmeasured confounding, estimates between two binary measures are needed (Ding and VanderWeele, 2016). Therefore, we used a positive cut-off of 1 z-score to define high *versus* low/moderate personality trait. Because there is no established cut-off value for this classification, we applied two sub-analyses i) setting 1.5 z-score as cut-off value, and ii) replicating the association analyses between medication use in pregnancy and personality traits as scores in numeric scale.

We used translated and validated versions of the BFI, whenever available. This was the case for the following languages: German, Italian, Norwegian, Polish, Slovenian, Spanish, and Swedish (Lupattelli et al., 2018b). Back-to-back translations of the BFI were carried out for the following languages: Croatian, Finnish, Russian, and Serbian.

### 2.3 Medication use in pregnancy

In the questionnaire, women were first presented a list of the most common acute/short-term illnesses (e.g., nausea, heartburn, sleeping problems) and the most prevalent chronic/long-term disorders (e.g., depression, anxiety) and asked whether they had these conditions during pregnancy. Women were then asked about medication use for each indication as free-text entry, along with the timing of usage (pregnancy weeks 0–12, 13–24, and 25–delivery). We defined a medicine as a single product containing one or more active ingredients, identified through relevant national medicine databases. We coded the recorded medicines into the corresponding Anatomical Therapeutic Chemical (ATC) Classification at the fifth level whenever possible, or otherwise into the second to fourth levels as appropriate, in accordance with the World Health Organization ATC index (“WHO Collaborating Centre for Drugs Statistics Methodology. ATC/DDD index, 2012”). Women reporting use in pregnancy of acetaminophen (ATC N02BE01, N02BE71, and N02BE51), opioid analgesics (ATC N02A), antidepressants (ATC group N06A), antipsychotics (ATC N05A), or anxiolytics and/or z-hypnotics (ATC N05BA, N05CD or N05CF) were considered as exposed to the medication groups of interest. Because extended use of acetaminophen has been linked specifically to adverse developmental outcomes in the offspring (Hjorth et al., 2019), we defined as extended users women reporting acetaminophen in all three trimesters of pregnancy. This was used as proxy of extended use, as the questionnaire lacks information on actual number of days the medication was taken.

### 2.4 Sociodemographic characteristics

Through the questionnaire, we collected information about maternal socio-demographics (i.e., region of residency, participant age, previous children, marital status, education level, employment situation), life-style factors (i.e., smoking status during pregnancy and alcohol consumption after awareness of pregnancy), and pregnancy status at the time of the research. Women also reported whether they had long-term conditions in pregnancy; those reporting depression, anxiety, bipolar disorder, schizophrenia or personality disorders were considered as having a pre-existing mental illness. We considered as possible confounders maternal age, having previous children, marital status, education level, employment situation, region of residency, and whether women were pregnant at the time of questionnaire response, as done in prior work (Lupattelli et al., 2018a).

### 2.5 Statistical analysis

We performed all statistical analysis using Stata MP 17.0. Descriptive statistics were conducted, and we applied survey weights based on the auxiliary variables age and education, which are important correlates of study response. National Statistics of the European Union, United States, Canada and Australia provided information about the distribution of age and education among women of childbearing age in each participating country. Each woman was assigned a weight, obtained by dividing the population proportion by the corresponding sample proportion in each age-by-education stratum. Women under-represented in our sample were assigned a weight greater than one, while those over-represented received a weight smaller than one. The survey weight for the entire study sample had a mean of 1.03 (range 0.1–55.0). To estimate the association between each personality dimension and use of the selected medications in pregnancy, we conducted unadjusted and adjusted logistic regression, using the survey weighting. Results are presented as odds ratio (OR) with 95% confidence interval (CI). In sensitivity analyses, linear regression models were fit when estimating association with personality traits as numeric z-scores. Results are presented as z-score mean difference β) with 95% confidence interval (CI). When examining the association between antidepressants, antipsychotics, benzodiazepines/z-hypnotics with maternal personality traits, we performed the analysis only in the stratum of women with a pre-existing mental illness, to assess the presence of an association between the medications and the personality trait independent of the mental illness.

Less than 1% of the women had missing values in any of the maternal factors (see Table 1), and up to 3.9% had missing information on at least one of the personality dimensions. Under the assumption that data were missing at random, we imputed incomplete data via multiple imputation with chained equation (20 imputed datasets) (Sterne et al., 2009; Perkins et al., 2018). Imputed data were used in all analyses.

**TABLE 1:** Baseline characteristics of the study population (*n* = 8879). Data are presented as number with percentages [n (%)] unless otherwise specified.

*Pregnancy characteristics*	
Pregnancy status at time of study participation	
Pregnant	4,806 (54.1)
Postpartum	4,073 (45.9)
Gestation week (range: 1–42); mean (sd)	22.5 (10.3)
Weeks since childbirth^a^	
0–12	875 (21.5)
13–24	1,007 (24.7)
≥ 25	2,191 (53.8)
Having prior children	
Yes	4,394 (49.5)
No	4,485 (50.5)

*Sociodemographic characteristics*	
Residence; no (%)	
Western Europe	3,038 (34.2)
Northern Europe	2,749 (31.0)
Eastern Europe	2,342 (26.4)
North America	533 (6.0)
Australia	217 (2.4)
Maternal age (in years, range 15–50); mean (sd)	29.7 (5.1)
Marital status; no (%)	
Married/cohabiting	8,352 (94.1)
Other than above	527 (5.9)
Working status at conception	
Employed in other sector than healthcare	5,297 (59.7)
Healthcare personnel	1,199 (13.5)
Student	770 (8.7)
Homemaker	762 (8.6)
Job seeker	403 (4.5)
None of the above	436 (4.9)
Missing	12 (0.1)
Educational attainment; no (%)	
< High school	346 (3.9)
High school	2,480 (27.9)
> High school	5,037 (56.7)
Other education	1,016 (11.4)

*Life-style characteristics*	
Alcohol use during pregnancy^b^	
Yes	1,444 (16.3)
No	7,353 (82.8)
Missing	82 (0.9)
Smoking during pregnancy	
Yes	845 (9.5)
No	8,012 (90.2)
Missing	22 (0.3)

*Medication use during pregnancy and mental illness*	
Antidepressants	
Yes	234 (2.6)
No	8,645 (97.4)
Benzodiazepines and z-hypnotics	
Yes	101 (1.1)
No	8,778 (8.9)
Antipsychotics	
Yes	67 (0.8)
No	8,812 (99.3)
Opioid analgesics	
Yes	245 (2.8)
No	8,634 (97.2)
Acetaminophen	
Yes	4827 (54.4)
No	4052 (45.6)
Acetaminophen, extended use	
Yes, all three trimesters	2,947 (33.2)
No	4,052 (45.6)
Pre-existing mental illness	
Yes	440 (5.0)

^a^ The proportions in percentage are calculated using the number of postpartum women as denominator (*n* = 4073).

^b^ Indicates alcohol consumption after awareness of the pregnancy.

#### 2.5.1 Unmeasured confounding assessment

We estimated the E-value based on prior methodological work (VanderWeele and Ding, 2017), and assessed the sensitivity of observed associations to uncontrolled confounding by maternal neuroticism in the context of one long-term safety study within our group (Lupattelli et al., 2021). The applied example was based on data from the nationwide Norwegian Mother, Father, and Child Cohort Study (MoBa) linked to the Norwegian Patient Registry (Magnus et al., 2016); it assessed the long-term reproductive safety of antidepressant exposure on child risk for ADHD (Lupattelli et al., 2021). The observed weighted hazard for ADHD at child age 7–9 years was 1.93 (95% CI 1.22–3.05) with prenatal antidepressant exposure relative to non-exposed pregnancies within women with depression/anxiety. Because ADHD is relatively rare (<15%), we used the E-value formula for risk ratios (that is, “RR + sqrt{RR x (RR-1)}” although our observed point estimates were hazard ratios (VanderWeele and Ding, 2017). As recommended, we computed the E-value for the observed point estimate and for the 95% confidence limit closest to the null.

## 3 Results

The study population included 8,879 women, which equals 92.3% of those who replied to the consent question (see Figure 1). In total, 4,806 (54.1%) women were pregnant at the time of questionnaire completion, and the remainder (n = 4,073, 45.9%) were mothers of a child with less of 1 year of age. Table 1 summaries the distribution of sociodemographic, lifestyle and maternal characteristics of the study population. Most participants were from European countries and were married/cohabiting. The average age of the participants was 29.7 years. A total of 440 women (5.0%) reported having mental illnesses prior to pregnancy. Overall, 4,827 (54.4%) women reported use of acetaminophen at least once during pregnancy, and 245 (2.8%) reported use of opioid analgesics. There were 2,947 (33.2%) women reporting use of acetaminophen in all three trimesters. Antidepressants (n = 234, 2.6%) were the most commonly used psychotropic medications, followed by benzodiazepines/z-hypnotics (n = 101, 1.1%), and antipsychotics (n = 67, 0.8%) (Table 1).

Table 2 shows the survey-weighted mean scores and 95% CI of maternal personality traits, overall and by type medication use in pregnancy. The weighted proportion of women with high personality traits, overall and by prenatal medication exposure, are presented in Supplementary Table S1. Women reporting use of antidepressants, benzodiazepines/z-hypnotics, opioid analgesics, or antipsychotics had higher neuroticism trait than non-users of these medications; the largest mean difference on the neuroticism trait was observed in relation to antidepressant and benzodiazepine/z-hypnotic use.

**TABLE 2:** Survey-weighted mean scores of personality traits, overall and by type of medication taken during in pregnancy (n = 8,879).

	Personality traits, mean (95% CI)				
	Extraversion	Agreeableness	Consciousness	Neuroticism	Openness
Total population	27.1 (26.9–27.3)	33.5 (33.3–33.7)	29.9 (29.7–30.1)	22.5 (22.3–22.7)	33.8 (33.6–34.0)
*Medication taken during pregnancy*					
*Antidepressants*					
Yes	24.5 (23.5–25.4)	32.5 (31.5–33.5)	27.9 (26.9–28.9)	27.9 (27.0–28.8)	32.6 (31.2–34.0)
No	27.2 (26.9–27.4)	33.6 (33.3–33.8)	30.0 (29.8–30.1)	22.4 (22.2–22.6)	33.9 (33.6–34.1)
*Benzodiazepines and z-hypnotics*					
Yes	26.4 (24.9–28.0)	31.5 (27.5–33.4)	28.0 (25.8–30.2)	28.2 (26.8–29.6)	33.4 (30.7–36.2)
No	27.1 (26.9–27.3)	33.5 (33.3–33.8)	29.9 (29.8–30.1)	22.5 (22.3–22.7)	33.8 (33.6–34.0)
*Antipsychotics*					
Yes	25.8 (24.1–27.4)	32.6 (31.1–34.1)	28.5 (26.9–30.1)	24.5 (23.0–26.0)	34.1 (32.6–35.5)
No	27.1 (26.9–27.3)	33.6 (31.1–34.1)	29.9 (29.9–30.1)	22.5 (22.3–22.7)	33.8 (33.6–34.0)
*Analgesic opioids*					
Yes	27.1 (26.3–27.8)	33.3 (32.6–34.1)	29.4 (28.7–30.1)	24.0 (23.1–24.9)	33.4 (32.6–34.1)
No	27.1 (26.9–27.3)	33.5 (33.3–33.7)	29.9 (29.8–30.1)	22.5 (22.3–22.7)	33.8 (33.6–34.1)
*Acetaminophen*					
Yes	26.9 (26.6–27.2)	34.0 (33.8–34.3)	30.1 (29.9–30.3)	22.6 (22.3–22.9)	33.4 (33.1–33.8)
No	27.4 (27.1–27.6)	32.9 (32.6–33.2)	29.8 (29.5–30.0)	22.5 (22.3–22.8)	34.3 (34.0–34.6)
*Acetaminophen, extended use*					
Yes, all three trimesters	26.6 (26.1–27.0)	34.0 (33.7–34.4)	29.9 (29.7–30.2)	22.7 (22.3–23.2)	33.2 (32.9–33.5)
No	27.4 (27.1–27.6)	32.9 (32.6–33.2)	29.8 (29.5–30.0)	22.5 (22.3–22.8)	34.3 (34.0–34.6)

The personality dimensions scores have range: 8–40 (extraversion and neuroticism), 9–45 (agreeableness and conscientiousness), 10–50 (openness).

Abbreviations: CI, confidence interval.

Table 3 describes the association between maternal personality and use of medication types in pregnancy. After adjusting for possible confounders, there was a strong association between having high neuroticism and prenatal use of antidepressants (adjusted OR: 5.63, 95% CI: 3.96–8.01), benzodiazepines and z-hypnotics (adjusted OR: 6.66, 95% CI: 4.05–10.95), and analgesic opioids (adjusted OR: 2.24, 95% CI: 1.41–3.56). In the stratum of women with long-term mental illness, the association was attenuated for benzodiazepines/z-hypnotics (adjusted OR: 2.24, 95% CI: 1.06–4.69), but decreased substantially for antipsychotics (adjusted OR: 0.48, 95% CI: 0.21–1.09) and antidepressants (adjusted OR: 0.74.95% CI: 0.45, 1.21).

**TABLE 3:** Association between high personality trait–defined as ≥1 z-score - and use of specific medications during in pregnancy (n = 8,879).

*Extraversion*		*Agreeableness*		*Consciousness*		*Neuroticism*		*Openness*	
cOR (95% CI)	aOR^a^ (95% CI)	cOR (95% CI)	aOR^a^ (95% CI)	cOR (95% CI)	aOR^a^ (95% CI)	cOR (95% CI)	aOR^a^ (95% CI)	cOR (95% CI)	aOR^a^ (95% CI)
*Antidepressants (yes vs. no)*									
0.66 (0.37–1.18)	0.77 (0.42–1.40)	0.71 (0.47–1.03)	0.63 (0.42–0.95)	0.58 (0.36–0.93)	0.58 (0.36–0.93)	5.04 (3.60–7.05)	5.63 (3.96–8.01)	0.95 (0.63–1.41)	1.18 (0.78–1.77)
*Benzodiazepines and z-hypnotics (yes vs. no)*									
0.89 (0.45–1.75)	0.97 (0.48–1.96)	0.63 (0.34–1.67)	0.63 (0.33–1.18)	1.35 (0.76–2.38)	1.49 (0.84–2.64)	6.93 (4.29–11.19)	6.66 (4.05–10.95)	1.21 (0.70–2.09)	1.31 (0.73–2.32)
*Antipsychotics (yes vs. no)*									
0.73 (0.29–1.81)	0.85 (0.32–2.23)	0.69 (0.34–1.43)	0.71 (0.35–1.46)	1.14 (0.42–3.11)	1.24 (0.40–3.86)	1.74 (0.97–3.14)	1.64 (0.87–3.12)	0.87 (0.41–1.81)	1.14 (0.51–2.53)
*Analgesic opioids (yes vs. no)*									
0.99 (0.67–1.47)	1.08 (0.73–1.60)	1.02 (0.70–1.47)	0.93 (0.64–1.36)	1.11 (0.76–1.62)	0.97 (0.67–1.41)	1.92 (1.23–3.00)	2.24 (1.41–3.56)	0.60 (0.38–0.94)	0.82 (0.52–1.28)
*Acetaminophen (yes vs. no)*									
0.88 (0.75–1.03)	1.00 (0.85–1.17)	1.43 (1.17–1.75)	1.31 (1.03–1.66)	0.91 (0.78–1.06)	0.85 (0.73–0.99)	0.99 (0.84–1.17)	1.11 (0.93–1.32)	0.78 (0.63–0.96)	0.97 (0.75–1.24)
*Acetaminophen, extended use (all three trimesters vs. no)*									
0.80 (0.67–0.97)	0.93 (0.78–1.12)	1.35 (1.09–1.67)	1.13 (0.94–1.37)	0.88 (0.74–1.05)	0.80 (0.67–0.96)	1.10 (0.92–1.33)	1.31 (1.08–1.59)	0.66 (0.55–0.79)	0.77 (0.64–0.93)

Abbreviations: CI, confidence interval; cOR, crude odds ratio; aOR, adjusted odds ratio.

^a^ Adjusted for maternal age, having previous children, marital status, education level, employment situation, region of residency, and whether women were pregnant at the time of questionnaire response, using the survey weights.

Having greater agreeableness was associated with 37% lower likelihood of using antidepressants in pregnancy, but no such association was seen with other psychotropics. High agreeableness and high conscientiousness were respectively associated with greater (31% increased) and lower (15% reduced) likelihood of using acetaminophen in pregnancy (Table 3). The former association reduced to 1.13 with a 95% CI crossing the null when considering extended use of acetaminophen in pregnancy. Having high neuroticism and high openness were respectively associated with 31% greater and 23% reduced likelihood of extended use of acetaminophen in pregnancy. Results of the sub-analysis using 1.5 z-score as cut-off value for high personality traits or modelling personality score as numeric scale, are presented in Supplementary Tables S2, S3, respectively and generally showed similar trends.

### 3.1 Applied example

In our applied example (Lupattelli et al., 2021), the observed point estimate was 1.93, with 1.22 as lower bound of the CI; based on these estimates, the corresponding E-values were respectively 3.27 and 1.74. Given the strength of the association between maternal neuroticism and antidepressant exposure in pregnancy found in this study (adjusted OR: 5.63, 95% CI: 3.96–8.01 if moderate neuroticism is defined as ≥1.0 z-score or adjusted OR: 5.46, 95% CI: 3.60–8.30 with cut-off at 1.5 z-score), and assuming equal strength of the unmeasured confounder with ADHD, high neuroticism in the mother could explain away the association antidepressant-ADHD. Because our example study compared antidepressant exposed with unexposed pregnancies within women with depression/anxiety, it is of relevance to examine unmeasured confounding by neuroticism beyond and above maternal mental illness. Given the null association between neuroticism and antidepressant use in pregnancy in the stratum of women with long-term mental illness (adjusted OR: 0.74.95% CI: 0.45, 1.21), unmeasured confounding by high neuroticism in the mother could not explain away the association antidepressant-ADHD identified among children born to women with a mental illness.

## 4 Discussion

This study reports new knowledge about the relationship between five maternal personality traits and prenatal use of antidepressants, antipsychotics, anxiolytics/z-hypnotics, analgesic opioids, and acetaminophen, including extended use of the latter. By providing robust estimates for the strength of these associations, this study can aid pharmacoepidemiological safety studies in pregnancy when appraising the bias due to uncontrolled confounding by maternal personality. As shown in our example on the association between prenatal antidepressants and child ADHD, setting plausible parameters for the association between the uncontrolled confounder and both exposure and outcome, can assist the overall appraisal of the study results. Yet, caution is needed in interpreting results in light of the E-value, to avoid both underestimation or overestimation of bias due to uncontrolled confounding (Sjölander and Greenland, 2022). Several findings are important for advances in real-world assessment of prenatal drug effects on offspring development, but also for clinical practice (e.g., including the assessment of maternal personality when counselling about pharmacological treatment in pregnant women).

We found that prenatal use of antidepressants, benzodiazepines/z-hypnotics, and analgesic opioids is strongly associated with high neurotic disposition in the woman, even after adjusting for well-known risk factors such as age, education, employment, marital status, parity, and region of residency. Women with high neurotic traits might be more vulnerable to mental disorders and require psychotropic treatment to a greater degree than other women. This finding is in line with prior genetic research (Fabbri et al., 2021; Andreassen et al., 2023) and is further supported by observational data collected in Norway in 2012, using a similar web-based approach (Ystrom et al., 2012). In this latter study (Ystrom et al., 2012), the associations measures between maternal neuroticism and antidepressants or benzodiazepines/z-hypnotics use in pregnancy were comparable to those observed in the current investigation, also in terms of effect size. This replication of result in the context of a lager, multinational scale study, corroborates the important role of high neuroticism in the pregnant woman in relation to use of specific psychotropics.

As expected, there was no longer any association between high neurotic disposition in the woman and use of antidepressants in the stratum of women with long-term mental illness. Based on this, it is reasonable to assume that uncontrolled confounding by high neurotic disposition in the mother would not bias our results for prenatal antidepressants and child ADHD, as this trait is highly correlated with mental illness which was controlled for, by restriction (Lupattelli et al., 2021). Assessing the reproductive long-term safety of prenatal medication exposures on offspring outcomes always requires as first step a methodologically sound design and well-informed analysis, including methods for triangulating the evidence and informative sensitivity analyses (Huybrechts et al., 2019). On top of this, it is crucial to be reminded that most pharmacoepidemiological pregnancy studies face the challenge of numerous unmeasured confounders, including genetic, and assessing their inter-related effects on the exposure and outcome of interest, remains difficult to quantify.

One key result is that the magnitude of the association between antipsychotic use in pregnancy and high neurotic disposition in the woman was not as high as for antidepressants or benzodiazepines/z-hypnotics, and the 95% CI of this association included the null. This is somewhat surprising as genetic studies have shown a correlation between higher neuroticism and psychosis (Boyette et al., 2013). We cannot exclude the possibility that our low prevalence for this medication exposure produced an unstable association measure with a broad CI. On the other hand, the association of this maternal trait with maternal use of benzodiazepines/z-hypnotics was elevated, and remained substantial also among those women having a long-term mental illness. This may suggest an association between neuroticism benzodiazepines/z-hypnotics independent of the maternal mental illness; indeed, these medications can be prescribed for multiple indications including insomnia and pain management (Larochelle et al., 2015). Nevertheless, this finding indicates that maternal neuroticism is possibly key confounder in pharmacoepidemiological studies assessing the long-term reproductive safety of benzodiazepines/z-hypnotics, and the E-value method should be used in addition to other methods for triangulation, to limit as much as possible this risk of bias.

Agreeableness and conscientiousness emerged as other traits related to medication use in pregnancy, albeit the effect sizes were modest. Both traits were inversely associated with antidepressant use in pregnancy. These results were not observed by Ystrom et al. (Ystrom et al., 2012). It is plausible that higher conscientiousness would be associated with a lower level of medication use, including antidepressants, as this may be proxy of risky health-related behaviour (Bogg and Roberts, 2004). Similarly, women with greater agreeableness may have higher trust in clinical recommendations and healthcare professionals. At the time of the data collection in this study back in 2011–2012, the available evidenced base about the reproductive safety of antidepressants was still limited (Spigset and Nordeng, 2016), possibly affecting clinical recommendations to women about pharmacological treatment of their mental illness.

Given the vivid research debate regarding the long-term safety of prolonged use of acetaminophen during pregnancy (Bauer et al., 2021; Alwan et al., 2022), it is of interest to evaluate the strength of association between this exposure and maternal personality traits. We found that high neurotic disposition in the woman was associated with a modest increased likelihood of extended use of acetaminophen in pregnancy, but for openness the association was the converse. Given the moderate strength of these associations, it is unlikely that uncontrolled confounding by maternal personality alone would cancel out the association between extended use of acetaminophen in pregnancy and child ADHD found in some studies (Hjorth et al., 2019). However, the role of other unmeasured confounders such as familial environment and genetics, has been shown to be substantial (Leppert et al., 2019). Taken together, these findings underscore the need of triangulating the evidence using different causally informative approaches and in light of possible biases introduced by numerous unmeasured confounders.

The study has various strengths and weaknesses. An important strength is that the data was collected from the same questionnaire across all participating countries, and we corrected our associations and descriptive estimates by survey weight adjustment, allowing the findings to be more generalizable in terms of age and education. Our use of a web-based questionnaire also ensured that we reached a large enough population to draw meaningful statistical inferences. Various studies have indicated that web-based recruitment methods give reasonable validity (Ekman and Litton, 2007; van Gelder et al., 2018; 2010). Another strength of the study is that the measurement we used for personality is a validated scale with good internal consistency, which is used in most studies about personality (John et al., 2008). This makes it easy to interpret the study’s results in light of other findings in the literature. The data we collected was also anonymous. This is important because perceived confidentiality and anonymity are important factors to counteract social desirability bias. We also imputed missing data on covariates, which is a methodological advantage (Sterne et al., 2009).

One weakness of the study is the selection bias in web-based sampling. The questionnaire was only available through internet websites, which did not permit calculation of a conventional response rate. Women who participated may also differ from those who did not in terms of sociodemographic characteristics, health status or internet access leading to potential selection bias. In general, the internet penetration rate, either in households or at work, is relatively high among women of childbearing age (73% households in the EU had internet access in 2011) (Seybert, 2011). However, the women who responded to our web-based questionnaires had higher education levels than the average birthing population, and were more often primiparous. To examine this weakness, we have previously compared the sociodemographic and lifestyle characteristics of the study sample to the population in the countries the study is based on (Lupattelli et al., 2014). Although we made our study more generalizable in terms of age and education via survey weighting, selection bias due to access to internet cannot be excluded. In addition, the cross-sectional design based on surveys may introduce recall bias as information on medication use was reported retrospectively. However, as personality trait is a time-fixed factor, poor retrospective recall is less likely to be present.

## Conclusion

The findings of this study show the importance of maternal personality disposition in relation to medication use during pregnancy. In general, women with high neurotic trait were more likely to use antidepressants, benzodiazepines/z-hypnotics, extended use of acetaminophen, or opioid analgesics during pregnancy. There was no strong evidence for this association in relation to antipsychotic use. Agreeableness, conscientiousness and openness emerged as other traits related to medication use in pregnancy, but had more mixed effects as they increased the likelihood of using some medications and decreasing others. The strength of these association can aid researchers in evaluating the influence of uncontrolled confounding by maternal personality in long-term safety studies in pregnancy, using the E-value. This assessment should be always be performed in addition to a rigorous study design using approaches to triangulate the evidence.

## Data Availability

The original contributions presented in the study are included in the article/Supplementary Material. Researchers can apply for data access for subprojects within the overall aims of the main study “The Multinational Medication Use in Pregnancy Study”, further inquiries can be directed to the corresponding author.
