# Supplementary material for: Association of maternal personality traits with medication use during pregnancy to appraise unmeasured confounding in long-term pharmacoepidemiological safety studies
Source: Front Pharmacol. 2023 May 15;14:1160168. doi: 10.3389/fphar.2023.1160168 (PMC10225644; doi:10.3389/fphar.2023.1160168)
Supplement: Supplementary file 1 [file DataSheet1.PDF]

Supplementary Material

Association of maternal personality traits with medication use during pregnancy to appraise unmeasured confounding in long-term pharmacoepidemiological safety studies

Angela Lupattelli*, Nhung TH Trinh, Hedvig Nordeng

***Correspondence:** Angela Lupattelli: angela.lupattelli@farmasi.uio.no

Supplementary Tables

Supplementary Table 1: Survey-weighted proportions of high personality traits, overall and by type of medication taken during pregnancy (n=8879)

	High personality traits, proportion% (95% CI)				
	Extraversion	***Agreeableness***	***Consciousness***	***Neuroticism***	***Openness***
Total population	14.1 (13.1-15.0)	17.0 (15.4-18.5)	13.4 (12.5-14.3)	16.1 (15.0-17.2)	16.0 (14.5-17.4)
Medication taken during pregnancy					
Antidepressants					
Yes	9.8 (4.7-15.0)	12.6 (8.3-16.8)	8.3 (4.7-11.8)	47.5 (39.4-55.6)	15.3 (10.3-20.2)
No	14.2 (13.2-15.2)	17.1 (15.5-18.7)	13.6 (12.6-14.5)	15.2 (14.1-16.3)	16.0 (14.5-17.4)
Benzodiazepines and z-hypnotics					
Yes	12.7 (5.2-20.2)	11.4 (5.2-17.6)	17.2 (9.2-25.2)	56.2 (44.6-67.8)	18.6 (10.5-17.4)
No	14.1 (13.1-15.0)	17.0 (15.5-18.6)	13.4 (12.5-14.3)	15.6 (14.5-16.7)	15.9 (14.5-17.4)
Antipsychotics					
Yes	10.7 (2.1-19.3)	12.4 (4.7-20.2)	15.0 (2.2-35.9)	25.0 (14.1-35.9)	14.1 (5.3-23.0)
No	14.1 (13.1-15.1)	17.0 (15.4-18.6)	13.4 (12.5-14.3)	16.0 (14.9-17.2)	16.0 (14.5-17.4)
Analgesic opioids					
Yes	13.9 (9.3-18.6)	17.2 (12.2-22.2)	14.6 (10.0-19.2)	26.5 (18.0-35.0)	10.3 (6.3-14.4)
No	14.1 (13.1-15.0)	17.0 (15.4-18.9)	13.3 (12.3-14.3)	15.8 (14.7-16.9)	16.1 (14.6-17.6)
Acetaminophen					
Yes	13.4 (12.1-16.6)	19.2 (16.7-21.8)	12.9 (11.7-14.1)	16.1 (14.6-17.5)	14.4 (12.2-16.7)
No	14.9 (13.4-16.4)	14.2 (12.9-15.6)	14.0 (12.7-15.4)	16.2 (14.4-17.9)	17.8 (16.2-19.4)
Acetaminophen, extended use					
Yes, all three trimesters	12.3 (10.8-13.9)	18.3 (15.6-21.0)	12.6 (11.1-14.0)	17.6 (15.6-19.5)	12.5 (10.9-14.0)
No use	14.9 (13.4-16.4)	14.2 (12.9-15.6)	14.0 (12.7-15.4)	16.2 (14.4-17.9)	17.8 (16.2-19.4)

Proportions were calculated using 1 z-score on the personality dimension as cutoff value.

Abbreviations: CI=Confidence Interval.

Supplementary Table 2: Association between high personality trait – defined as ≥1.5 z-score - and use of specific medications during in pregnancy (n=8879)

Extraversion		*Agreeableness*		*Consciousness*		*Neuroticism*		*Openness*	
**cOR (95% CI)**	**aOR (95% CI)**	**cOR (95% CI)**	**aOR (95% CI)**	**cOR (95% CI)**	**aOR (95% CI)**	**cOR (95% CI)**	**aOR (95% CI)**	**cOR (95% CI)**	**aOR (95% CI)**
Antidepressants (yes vs. no)									
0.17 (0.05-0.55)	**0.22 (0.07-0.71)**	0.75 (0.40-1.41)	0.63 (0.33-1.21)	0.59 (0.27-1.29)	0.63 (0.29-1.37)	4.52 (3.09-6.62)	5.46 (3.60-8.30)	1.24 (0.74-2.07)	1.56 (0.92-2.65)
BZDs and z-hypnotics (yes vs. no)									
0.54 (0.12-2.25)	0.60 (0.15-2.51)	-	-	1.29 (0.55-3.05)	1.45 (0.62-3.39)	5.59 (3.31-9.43)	5.19 (2.88-9.36)	1.37 (0.68-2.76)	1.49 (0.72-3.08)
Antipsychotics (yes vs. no)									
-	-	1.02 (0.36-2.93)	0.86 (0.28-2.68)	-	-	2.04 (0.99-4.17)	1.93 (0.87-4.31)	1.37 (0.56-3.35)	1.66 (0.63-4.37)
Analgesic opioids (yes vs. no)									
0.69 (0.35-1.36)	0.91 (0.46-1.82)	0.98 (0.55-1.75)	0.84 (0.46-1.53)	0.86 (0.49-1.48)	0.82 (0.47-1.42)	1.61 (1.05-2.48)	1.99 (1.25-3.17)	0.56 (0.30-1.04)	0.74 (0.40-1.39)
Acetaminophen (yes vs. no)									
0.77 (0.60-1.00)	0.95 (0.74-1.21)	1.63 (1.17-2.28)	1.34 (0.99-1.83)	0.76 (0.62-0.93)	**0.75 (0.61-0.91)**	0.86 (0.69-1.08)	1.00 (0.79-1.26)	0.71 (0.57-0.87)	0.88 (0.71-1.09)
Acetaminophen, extended use (all three trimesters vs. no)									
0.69 (0.52-0.93)	0.84 (0.64-1.12)	1.72 (1.12-2.64)	1.30 (0.91-1.85)	0.65 (0.52-0.82)	0.62 (0.49-0.78)	0.97 (0.76-1.24)	1.15 (0.88-1.49)	0.64 (0.50-0.81)	0.78 (0.60-0.98)

Results are not shown when counts are <3.

Abbreviations: CI=Confidence Interval, cOR=Crude Odds Ratio; aOR=Adjusted Odds Ratio.

[a]Adjusted for maternal age, having previous children, marital status, education level, employment situation, region of residency, and whether women were pregnant at the time of questionnaire response, using the survey weights.

Supplementary Table 3: Association between personality trait – defined as numeric z-score - and use of specific medications during in pregnancy (n=8879)

Extraversion		***Agreeableness***		***Consciousness***		***Neuroticism***		***Openness***	
**Crude β (95% CI)**	**Adjusted β (95% CI)**	**Crude β (95% CI)**	**Adjusted β (95% CI)**	**Crude β (95% CI)**	**Adjusted β (95% CI)**	**Crude β (95% CI)**	**Adjusted β (95% CI)**	**Crude β (95% CI)**	**Adjusted β (95% CI)**
Antidepressants (yes vs. no)									
-0.47 (-0.63,-0.30)	-0.36 (-0.53,-0.18)	-0.20 (-0.40,0.00)	-0.27 (-0.47,-0.08)	-0.41 (-0.61,-0.21)	-0.38 (-0.58,-0.18)	0.94 (0.78,1.10)	0.98 (0.81,1.15)	-0.20 (-0.42,0.02)	-0.13 (-0.36,0.10)
BZDs and z-hypnotics (yes vs. no)									
-0.11 (-0.39,0.16)	-0.06 (-0.34,0.22)	-0.41 (-0.80,-0.02)	-0.39 (-0.75,-0.01)	-0.38 (-0.82,0.05)	-0.33 (-0.75,0.09)	0.97 (0.74,1.21)	0.94 (0.70,1.17)	-0.06 (-0.49,0.37)	-0.03 (-0.47,0.41)
Antipsychotics (yes vs. no)									
-0.23 (-0.52,0.06)	-0.07 (-0.35,0.21)	-0.19 (-0.48,0.11)	-0.19 (-0.48,0.09)	-0.29 (-0.61,0.04)	-0.18 (-0.54,0.18)	0.34 (0.09,0.59)	0.30 (0.03,0.57)	0.04 (-0.19,0.27)	0.12 (-0.15,0.39)
Analgesic opioids (yes vs. no)									
-0.01 (-0.14,0.123	0.07 (-0.06,0.20)	-0.04 (-0.19,0.11)	-0.16 (-0.30,-0.01)	-0.11 (-0.25,0.03)	-0.15 (-0.29,-0.02)	0.25 (0.10,0.41)	0.34 (0.20,0.49)	-0.08 (-0.20,0.04)	0.02 (-0.09,0.14)
Acetaminophen (yes vs. no)									
-0.08 (-0.15,-0.01)	-0.01 (-0.07,0.05)	0.23 (0.15,0.31)	0.14 (0.05,0.23)	0.06 (-0.00,0.12)	0.02 (-0.04,0.09)	0.01 (-0.05,0.01)	0.07 (0.01,0.14)	-0.13 (-0.20,-0.06)	-0.04 (-0.11, 0.03)
Acetaminophen, extended use (all three trimesters vs. no)									
-0.14 (-0.23,-0.05)	-0.05 (-0.12,0.02)	0.23 (0.14,0.31)	0.10 (0.02,0.18)	0.04 (-0.04,0.11)	-0.02 (-0.10,0.05)	0.04 (-0.04,0.12)	0.12 (0.04,0.20)	-0.16 (-0.23,-0.10)	-0.08 (-0.14,-0.01)

Abbreviations: CI=Confidence Interval.

The β indicates the difference in z-score on the personality trait between the medication group users and non-users.

[a]Adjusted for maternal age, having previous children, marital status, education level, employment situation, region of residency, and whether women were pregnant at the time of questionnaire response, using the survey weights.
